# Differential levels of p75NTR ectodomain in CSF and blood in patients with Alzheimer's disease: a novel diagnostic marker

**DOI:** 10.1038/tp.2015.146

**Published:** 2015-10-06

**Authors:** S-S Jiao, X-L Bu, Y-H Liu, Q-H Wang, C-H Liu, X-Q Yao, X-F Zhou, Y-J Wang

**Affiliations:** 1 Department of Neurology, Daping Hospital, Third Military Medical University, Chongqing, China; 2 Division of Health Sciences, School of Pharmacy and Medical Sciences, Sansom Institute, University of South Australia, Adelaide, SA, Australia

## Abstract

Alzheimer's disease (AD) is the primary cause of dementia in the elderly. The ectodomain of p75 neurotrophin receptor (p75NTR-ECD) has been suggested to play important roles in regulating beta-amyloid (Aβ) deposition and in protecting neurons from the toxicity of soluble Aβ. However, whether and how the serum and cerebrospinal fluid (CSF) levels of p75NTR-ECD change in patients with AD are not well documented. In the present study, we determined the concentrations of serum p75NTR-ECD in an AD group, a Parkinson disease group and a stroke group, as well as in a group of elderly controls without neurological disorders (EC). We also determined the levels of CSF p75NTR-ECD in a subset of the AD and EC groups. Our data showed that a distinct p75NTR-ECD profile characterized by a decreased CSF level and an increased serum level was present concomitantly with AD patients but not with other diseases. p75NTR-ECD levels in both the serum and CSF were strongly correlated with Mini-Mental State Examination (MMSE) scores and showed sound differential diagnostic value for AD. Moreover, when combining CSF Aβ42, CSF Aβ42/40, CSF ptau181 or CSF ptau181/Aβ42 with CSF p75NTR-ECD, the area under the receiver operating characteristic curve (AUC) and diagnostic accuracies improved. These findings indicate that p75NTR-ECD can serve as a specific biomarker for AD and the determination of serum and CSF p75NTR-ECD levels is likely to be helpful in monitoring AD progression.

## Introduction

Alzheimer's disease (AD) is the most common form of neurodegenerative disease and is the primary cause of dementia in the elderly.^1, 2^ Several lines of evidence indicate that the p75 neurotrophin receptor (p75NTR) plays multiple roles in the pathogenesis of AD, including those related to beta-amyloid (Aβ) generation,^3^ neuronal death,^4, 5^ neuritic dystrophy^6^ and Tau hyperphosphorylation.^7, 8^ Our previous study found that the ectodomain of p75NTR (p75NTR-ECD), which is shed by the tumour necrosis factor-alpha-converting enzyme (TACE), inhibits Aβ aggregation and reduces the Aβ burden in the hippocampus of AD mice and that p75NTR-ECD deletion conversely increases the Aβ burden and AD phenotypes in mice.^9^ Soluble p75ECD has been suggested to be physiologically distributed in the central nervous system and is developmentally regulated by ageing. The level of p75NTR-ECD is significantly reduced in the brains of AD subjects and mice, likely due to the Aβ-induced reduction in the expression and activity of TACE.^10^ These findings suggest that p75NTR-ECD could be one of the endogenous protective mechanisms underlying the regulation of Aβ pathologies and that it likely plays an important role in preventing Aβ-induced neurotoxicity by protecting neurons from the toxicity of soluble Aβ and Aβ oligomers.^11^ However, to date, whether and to what extent the serum and cerebrospinal fluid (CSF) levels of soluble p75NTR-ECD change in patients with AD are not well documented, and the association of changes between p75NTR-ECD and cognition is also unknown.

Accordingly, we determined the concentrations of p75NTR-ECD in the serum of four groups of subjects including an AD group, Parkinson disease (PD) group (as a neurodegeneration disease control), an acute ischemic stroke group (as a cerebrovascular disease control) and age-matched elderly controls without neurologic disorders (EC), as well as the concentrations of CSF p75NTR-ECD in a subset within the AD and EC groups. We also detected Aβ40, Aβ42, total tau and phosphorylated tau (ptau181) levels in the serum and CSF of the AD and EC groups. We further investigated the association between cognitive functions and the level of p75NTR-ECD in the CSF and serum and then explored the utility of serum/CSF p75NTR-ECD alone or combined with other AD biomarkers for discriminating AD from other diseases.

## Materials and methods

### Subjects

This study was carried out on patients with AD, PD, acute ischemic stroke and EC. Participants with AD (*n*=156), PD (*n*=79) and stroke (*n*=83), and EC (*n*=129) were recruited from the department of neurology and the health examination centre of Daping Hospital. All AD subjects participating in this study met the clinical diagnostic criteria for probable AD according to the National Institute of Neurological and Communicative Disorders and Stroke–Alzheimer's disease and Related Disorders Association and DSM-IV criteria for dementia of the Alzheimer type. PD diagnosis was on the basis of the UK PD Society Brain Bank clinical diagnostic criteria, and each patient with PD was diagnosed by at least two specialists with >4 years of clinical experience on movement disorders. Patients with first-ever acute ischemic stroke were fully conscious and could cooperate with investigators to complete neurologic and neuropsychological examinations. The elderly controls consisted of subjects who had no history or signs of neurologic disorders in the clinical examination. The subjects per group were not eligible if they had (1) severe cardiac, pulmonary, hepatic or renal diseases or any type of tumour or (2) declined to participate in the study.

Clinical data were collected, including age, education, gender, comorbidities (for example, hyperlipidaemia, hypertension and diabetes mellitus) and the scores of multiple scales, such as mini-mental state examination (MMSE), clinical dementia rating (CDR) and activities of daily living (ADL). The scales mentioned above were conducted following our previous protocols,^12, 13^ and the evaluation on PD patients was performed in the clinical 'on' state. The study was approved by the Institutional Review Board of Daping Hospital and registered in the Chinese Clinical Trial Registry (No. ChiCTR-OCC-12001966). Informed consent was acquired from all subjects or, if needed, from authorized caregivers.

### Sample collection

Blood samples were collected from fasting participants and then centrifuged at 2000 *g* at 4 °C for 10 min. Serum was separated, and aliquots were stored at −80 °C until assayed. Some of the subjects underwent lumbar puncture (AD, *n*=25; EC, *n*=26). The CSF samples were centrifuged at 2000 *g* at 4 °C for 10 min to eliminate cells, and the aliquots were then immediately frozen and stored at −80 °C until biochemical analyses.

### ApoE genotyping

ApoE genotyping in AD and elderly control groups was performed via EDTA blood samples. ApoE genotypes (rs429358 and rs7412) were determined with the restriction fragment length polymorphism method. The PCR reactions were performed with 1 μl DNA sample, 1 × GC-I buffer (TaKaRa, Kusatsu, Japan), 2.0 mM Mg^2+^, 0.2 mM dNTP (Generay Biotech, Shanghai, China), 1U HotStarTaq polymerase (Qiagen, Hilden, Germany), 2 μl multiple PCR primers (Sangon, Shanghai, China) and ddH_2_O in a total volume of 10 μl. The cycling program was the same as described previously.^14^ The digestion of endonuclease was performed with AflIII or HaeII (New England Biolabs, Ipswich, MA, USA) for rs429358 or rs7412, respectively. Then, the products were analyzed with an ABI3130XL sequencer (Applied Biosystems, Waltham, MA, USA).

### ELISA assays of Aβ40, Aβ42, total tau, ptau181 and p75NTR-ECD

Enzyme-linked immunosorbent assay (ELISA) kits were used to determine the serum and CSF levels of Aβ40 (SIG-38950, Covance, Emeryville, CA, USA), Aβ42 (SIG-38953, Covance), total tau (KUB0041, Invitrogen, Waltham, MA, USA) and ptau181 (KUO0631, Invitrogen) according to the manufacturer's instructions. For Aβ measurement, serum and CSF samples were diluted to 1:24 and 1:3, respectively. For tau measurement, both serum and CSF samples were diluted to 1;1. To detect the serum and CSF levels of p75NTR-ECD, a human nerve growth factor receptor ELISA kit (R&D, Minneapolis, MN, USA) was used. Briefly, MaxiSorp 96-well plates were coated overnight with 2.0 μg ml^−1^ to capture the antibody at room temperature (RT) and then incubated with blocking buffer (5% bovine serum albumin in phosphate-buffered saline with 1% Tween-20) for 2 h at RT. Standards and samples (1:3 dilution) were prepared, and 100 μl of the standards or samples were added into the wells and then incubated for 2 h at RT. After washing, the plates were treated with 100 ml per well detection antibody (biotinylated goat anti-human nerve growth factor receptor IgG) at 200 ng ml^−1^ in blocking buffer for 1 h at RT. The plates were washed again and incubated with 100 ml per well Streptavidin-horseradish peroxidase in a 1:200 dilution for 1 h at RT. After a final wash, colour was developed via tetramethylbenzidine for 30 min. The plates were read at 450 nm using a microplate reader. The concentration of p75NTR-ECD was determined from a standard curve based on reference standards. The lowest detection limit was 1 pg ml^−1^. For all ELISA tests, samples and standards were measured in duplicate, and the means of the duplicates were used for statistical analyses.

### Statistical analysis

Data analyses were performed using SPSS (version 16, IBM, Armonk, NY, USA). The continuous variables were first assessed for normal distribution using the one-sample Kolmogorov–Smirnov test. Statistical comparisons of continuous variables among multiple groups were tested using a one-way analysis of variance test (for normal distributions) or a nonparametric Kruskal–Wallis test (for a non-normal distribution) as appropriate. Comparisons of continuous variables between two groups were performed using the independent-sample *t*-test or Mann–Whitney *U*-test, as applicable. The comparisons of categorical variables between groups were performed using Fisher's exact test or the *χ*^2^-test. The AD patients were divided into three subsets according to their MMSE scores: MMSE⩽10, MMSE=11–20 and MMSE⩾21, and the trend test was performed. Correlations between the p75NTR-ECD level and the scale scores and levels of Aβ and tau were analysed with Pearson or Spearman correlation analysis and partial correlation analysis with adjustment for age, education, sex and comorbidities. Optimal sensitivity and specificity were determined via the receiver operating characteristic (ROC) curve analysis utilising a nonparametric approach. The Youden index was calculated for each cutoff value as the corresponding [(sensitivity+specificity)−1] to determine the cutoff values that maximised the discriminating power of the test. The results are expressed as means±s.d. The statistical threshold was fixed at *P*<0.05.

## Results

### Characteristics of the study subjects

The details of demographic and clinical data of subjects with serum samples in the four groups are summarized in Table 1. There were no significant differences in age, education, sex, hyperlipidaemia or hypertension between the four groups. Consistent with previous studies,^15^ there was a higher occurrence of diabetes in the AD group compared with the other groups. As expected, AD patients had significantly lower MMSE scores and higher CDR and ADL scores than the other groups. The distribution of the ApoEɛ4 allele between AD and EC was significantly different. Additionally, the levels of four serum biomarkers (Aβ42, Aβ40, total tau and ptau181) in the AD group were significantly higher than their counterparts in the EC group.

The demographic and clinical information (including CSF levels of Aβ42, Aβ40, total tau and ptau181) of subjects with CSF samples (AD, *n*=25; EC, *n*=26) are presented in Supplementary Table 1. There were no differences in age, sex, comorbidities or ApoEɛ4 allele state between the two subset groups. Compared with EC participants, AD patients had lower education (*P*=0.01) and MMSE score (*P*<0.001). In terms of CSF biomarkers, AD patients had higher levels of total tau and ptau181 and lower levels of Aβ42 than the EC participants. The CSF levels of Aβ40 in AD were comparable with that in the EC group.

### p75NTR-ECD levels in the serum and CSF of different groups

**Figure 1:**
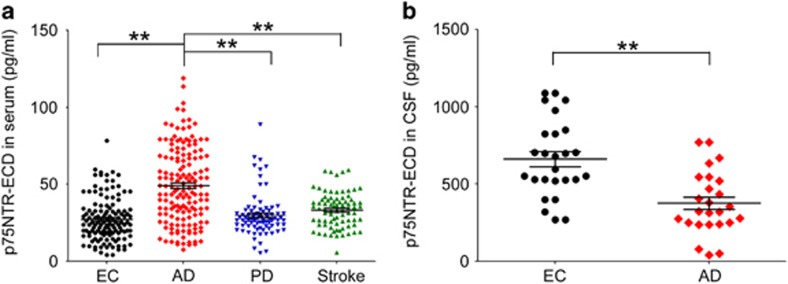
Ectodomain of p75 neurotrophin receptor (p75NTR-ECD) levels of serum and cerebrospinal fluid (CSF) in different groups. (**a**) Comparison of serum p75NTR-ECD levels among EC, AD patients, PD patients and stroke patients [means±s.e.m., one-way analysis of variance (ANOVA), Tukey's test, **P<0.01]. (**b**) Comparison of CSF p75NTR-ECD levels between EC and patients with AD (means±s.e.m., Student's *t-*test, **P<0.01). AD, Alzheimer's disease; EC, elderly controls without neurologic disorders; PD, Parkinson disease.

There was a significant difference in the serum levels of p75NTR-ECD among the groups (F=22.211, *P*<0.001; Figure 1a). *Post hoc* tests revealed that the serum p75NTR-ECD level in the AD group (49.14±24.53 pg ml^−1^) was significantly higher than that in the EC (29.36±20.21 pg ml^−1^), PD (33.36±11.35 pg ml^−1^) and stroke (35.17±25.39 pg ml^−1^) groups. No difference in the serum p75NTR-ECD level was found between EC and PD (*P*=0.571), EC and stroke (*P*=0.228) or PD and stroke (*P*=0.951). The CSF p75NTR-ECD level of the AD group (375.6±202.2 pg ml^−1^) was significantly lower than that of the EC group (661.7±248.2 pg ml−1; Figure 1b).

### Correlation of p75NTR-ECD with Aβ and tau

**Figure 2:**
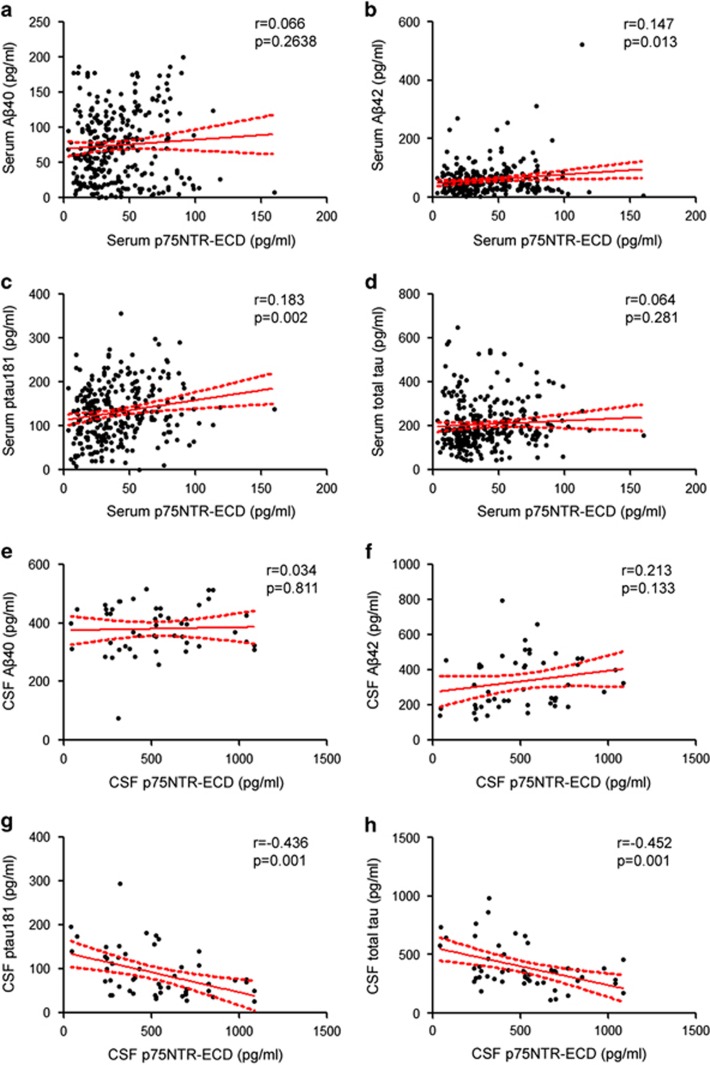
The correlation analyses of serum or cerebrospinal fluid (CSF) ectodomain of p75 neurotrophin receptor (p75NTR-ECD) with other biomarkers. (**a**–**d**) The correlation of serum p75NTR-ECD with serum Aβ40 (**a**), serum Aβ42 (**b**), serum ptau181 (**c**) and serum total tau (**d**). (**e**–**h**) The correlation of CSF p75NTR-ECD with CSF Aβ40 (**e**), CSF Aβ42 (**f**), CSF ptau181 (**g**) and CSF total tau (**h**).

The results of correlation analyses of p75NTR-ECD with Aβ40, Aβ42, ptau181 and total tau are shown in Figure 2. The serum p75NTR-ECD was positively correlated with serum Aβ42 (*r*=0.147, *P*=0.013) and serum ptau181 (*r*=0.183, *P*=0.002), and CSF p75NTR-ECD was negatively correlated with CSF ptau181 and total tau.

### Correlation of p75NTR-ECD with severity of AD

The correlation of serum p75NTR-ECD levels with the MMSE, CDR and ADL scores was investigated. Results are shown in Table 2. In a model including AD patients and EC subjects, statistical analysis revealed a remarkable negative correlation between the serum p75NTR-ECD level and MMSE scores (*r*=−0.472, *P*<0.001). After adjusting for age, education, sex and comorbidities, the correlation remained significant (*r*_adjusted_=−0.482, *P*_adjusted_<0.001). A similar result was also found for the correlation between the serum p75NTR-ECD level and CDR scores (*P*<0.001, *P*_adjusted_<0.001), indicating that a higher serum level of p75NTR-ECD was associated with a greater cognitive decline. The serum level of p75NTR-ECD was also positively correlated with ADL scores (*P*<0.001), suggesting that the serum p75NTR-ECD level may reflect the daily living disability. In another model including AD patients only, the serum level of p75NTR-ECD was also significantly correlated with MMSE, CDR and ADL scores (Table 2).

The CSF p75NTR-ECD level was also correlated with MMSE scores. The lower the CSF p75NTR-ECD level, the higher the severity of cognitive decline (*r*=0.608, *P*<0.001; *r*_adjusted_=0.554, *P*_adjusted_<0.001).

To further investigate whether serum p75NTR-ECD could be used as a predictor of AD progression, we divided AD patients into three subsets according to the MMSE score: MMSE⩽10, MMSE=11–20 and MMSE⩾21, and compared the difference in the p75NTR-ECD level between these three subsets. We found that the serum p75NTR-ECD concentration in the subset of MMSE⩽10 was significantly higher than that in MMSE=11–20 (*P*<0.001) or MMSE⩾21 (*P*<0.001; Supplementary Figure 1A). The AD patients in the subset with MMSE⩽10 also had a relatively lower CSF p75NTR-ECD level compared with AD patients with MMSE=11–20 (Supplementary Figure 1B). Trend tests further revealed that, with an increase in MMSE, serum and CSF p75NTR-ECD had a downward and upward trend, respectively (for serum, F=4.273, *P*<0.027; for CSF, F=18.093, *P*<0.001).

### Evaluation of p75NTR-ECD as a diagnostic marker for AD

**Figure 3:**
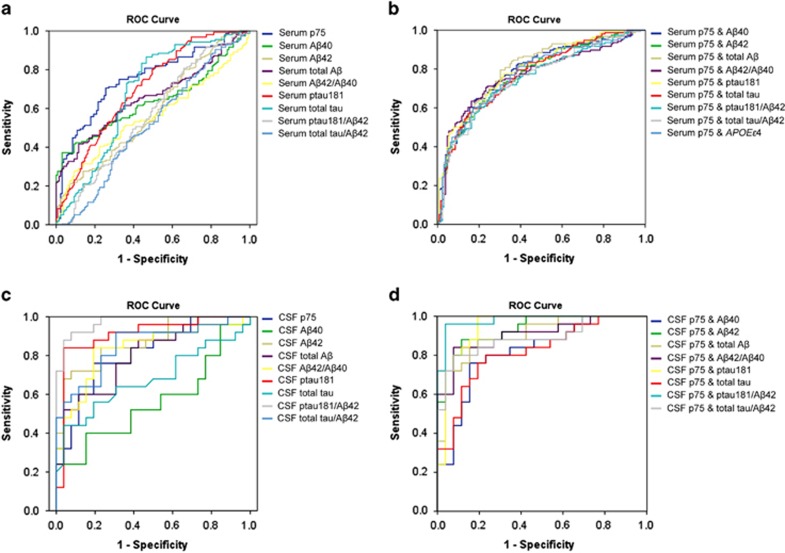
Receiver operating characteristic (ROC) curve of serum and cerebrospinal fluid (CSF) ectodomain of p75 neurotrophin receptor (p75NTR-ECD) alone or in combination with other biomarkers. (**a**) The ROC curve of serum p75NTR-ECD and other serum biomarkers. (**b**) The ROC curve of serum p75NTR-ECD in combination with other biomarkers. (**c**) The ROC curve of CSF p75NTR-ECD and other CSF biomarkers. (**d**) The ROC curve of CSF p75NTR-ECD in combination with other CSF biomarkers.

Given the correlation between p75NTR-ECD and cognitive decline in AD, we investigated whether the measurement of p75NTR-ECD is helpful in diagnosing AD. First, the discriminative value of serum p75NTR-ECD was evaluated. Using the ROC curve analysis, we found that the area under the ROC curve (AUC) of serum p75NTR-ECD in AD versus non-AD (including PD patients, stroke patients and EC subjects) was 0.720 (*P*<0.001, 95% confidence interval=0.666–0.774; Table 3). When applying the optimal cutoff value of 41.5 pg ml^−1^ calculated using the Youden index, the overall sensitivity and specificity of serum p75NTR-ECD for distinguishing AD from non-AD was 63.4% and 76.3%, respectively. Taken separately, the accuracy of serum p75NTR-ECD in AD versus EC, AD versus PD, and AD versus stroke was 72.5%, 75.3% and 73.7%, respectively. Notably, the specificity of serum p75NTR-ECD in AD versus PD was above 90% (Table 3). The AUC for serum p75NTR-ECD in AD versus EC was 0.752 (95% confidence interval=0.695–0.808), which was higher than any of the AUC values generated by each of the known serum biomarkers (Aβ42, Aβ40, total Aβ, Aβ42/Aβ40, ptau181, total tau, ptau181/Aβ42 and total tau/Aβ42; Supplementary Table 2). When combining each of these known serum biomarkers with serum p75NTR-ECD, the AUC value, sensitivity and specificity were all elevated compared with each alone (Supplementary Table 3; Figure 3a and b), indicating that serum p75NTR-ECD has a potential diagnostic value for AD when applied alone or in combination with serum Aβ and tau.

The utility of CSF p75NTR-ECD in discriminating AD from EC was also evaluated. The AUC for CSF p75NTR-ECD reached 0.815 (*P*<0.001, 95% confidence interval=0.700–0.931), and the sensitivity and specificity were 80.0% and 80.8%, respectively. When combining CSF p75NTR-ECD with CSF Aβ42, CSF Aβ42/40, CSF ptau181 or CSF ptau181/ Aβ42, the AUCs were above 0.900 (Figure 3c and d), and the accuracies were above 88% (Supplementary Table 3).

## Discussion

The role of p75NTR in AD pathogenesis has been well investigated; however, there are few studies focusing on the potential effects of p75NTR-ECD in AD. In the present study, we determined the concentrations of serum p75NTR-ECD in AD patients, PD patients, stroke patients and in EC, and found that serum p75NTR-ECD is specifically elevated in the AD group, indicating that p75NTR-ECD may benefit in diagnosing AD. Using a regression analysis, we then found that the level of p75NTR-ECD in the serum was strongly correlated with MMSE and CDR scores and had an important differentiating diagnostic value for AD. Moreover, within a subset cohort from which CSF samples were collected, we found that the level of p75NTR-ECD in the CSF was reduced in AD groups compared with elderly controls. Our data suggest that measurements of serum and CSF p75NTR-ECD may be helpful in AD diagnosis.

p75NTR-ECD, a soluble and diffusible molecule, is physiologically shed by TACE,^16^ and its shedding is considered to be a critical switch event that determines the balance between the enhancement effect on Aβ production and neurotoxicity of full-length p75NTR and the potential beneficial effect on blocking the toxicity of the Aβ oligomer of the p75NTR-ECD.^9, 10^ Thus, p75NTR-ECD may be an important modulating molecule in AD pathogenesis. In the present study, we found for we believe the first time that, compared with age-matched controls, only the AD group demonstrated a higher serum p75NTR-ECD level among three disease groups (AD, PD and stroke), indicating that this change was likely to be AD-specific. Why the p75NTR-ECD level in serum was elevated in AD is not known. It may be due to the alteration of plasma TACE activity. Plasma TACE activity in subjects with mild cognitive impairment and patients with AD is known to be increased.^17, 18, 19^ Because TACE is the main cleaving enzyme of p75NTR,^20, 21^ it is reasonable to suggest that a higher TACE activity would generate a higher level of p75NTR-ECD in the serum in AD. p75NTR is not only expressed in the central nervous system but also in the peripheral nervous system,^22^ liver,^23^ skeletal muscle,^24^ white adipose tissue^25^ and lymphocytes.^26^ Considering that Aβ can trigger the upregulation of p75NTR,^27, 28^ it is possible that the increased Aβ in the periphery upregulates the expression of p75NTR in these peripheral tissues, which would thereby increase the circulating p75NTR-ECD level in parallel with the full-length p75NTR. Whether the full-length p75NTR in blood cells is increased in AD requires further investigation. Paradoxically, we also found a reduction in CSF p75NTR-ECD in AD patients. Aβ and other neurodegenerative ligands, such as prion protein, are known to induce the endocytosis of TACE and reduction in TACE activity via PDK1 activation in the AD brain.^29^ Aβ accumulation in the AD brain could conceivably reduce TACE activity, which leads to the reduction of p75NTR shedding and the reduction of the p75NTR-ECD level in the CSF of AD subjects.^10^

The findings of the present study showed a parallel change in p75NTR-ECD and Aβ in the CSF and serum of AD subjects. Soluble Aβ40 and 42 are well known to be decreased in CSF^18, 19^ and increased in the peripheral blood,^30^ which represent changes that are used for diagnosing AD.^31, 32^ We found here that the p75NTR-ECD level is also reduced in the CSF and increased in the serum, and that both phenomena have diagnostic significance and are sensitive and specific as Aβ. This discovery is not only significant for determining AD diagnosis but also links the neurotrophic hypothesis with the amyloid hypothesis in AD. The mechanism underlying the parallel changes in Aβ and p75NTR-ECD remains unclear; however, p75NTR-ECD can bind with Aβ,^33^ and both full-length p75NTR and p75NTR-ECD affect Aβ metabolism.^3, 9^ Thus, these two events may be interdependent in AD pathogenesis. Here, we propose that the aberrant metabolism of p75NTR-ECD in CSF may disrupt the steady state of Aβ in CSF and subsequently promote the abnormal deposition of Aβ in brains and, as a result, decrease the soluble Aβ in the CSF. On the other hand, the overproduction of Aβ in the AD brain interferes with the shedding of p75NTR-ECD, resulting in a reduced amount of p75NTR-ECD in CSF. To date, the mechanism linking Aβ with tau remains unclear. In the present study, we found that p75NTR-ECD is significantly correlated with ptau181 both in the serum and CSF. In light of the role of p75NTR-ECD in Aβ metabolism, this phenomenon may imply that p75NTR-ECD is a mediator linking Aβ with tau. Taken together, p75NTR-ECD provides a novel perspective into AD pathogenesis. This perspective indicates that p75NTR-ECD is a promising therapeutic target and diagnostic marker for AD.

Although knowledge on neuroimaging biomarkers has greatly increased,^34^ biomarkers from the serum or CSF are also required to improve the diagnostic sensitivity and specificity and monitor AD progression.^35^ Our data showed that p75NTR-ECD levels are strongly associated with MMSE and CDR scores, implying that p75NTR-ECD, as a novel biomarker, may be useful in distinguishing AD from other diseases. Applying the Youden index method, we found that the serum p75NTR-ECD level had a diagnostic accuracy of ~70%, and that CSF p75NTR-ECD had an accuracy of ~80%. The application of p75NTR-ECD alone or in combination with other biomarkers can be used to strongly supplement classical biochemical and neuroimaging markers in the diagnosis of AD. Of note, because p75NTR-ECD correlates with the severity of AD, measurements of p75NTR-ECD levels may be of great use in monitoring AD progression in clinical trials and could be used as an early predictor for the progression from prodromal to AD-type dementia.

Novel biomarkers for AD have been continuously emerging. N-truncated forms of Aβ in CSF, such as Aβ 11–40, Aβ 11–42, Aβ 17–40 and Aβ 17–42, have been suggested to have potential diagnostic value in early AD detection and mild cognitive impairment stratification.^36, 37^ In contrast to these studies, the present study did not focus on the early AD stage, which is widely considered to be a pivotal time for disease-modifying therapies of AD.^38, 39^ Our future efforts will focus on determining whether serum and CSF p75NTR-ECD levels are predictors of AD in prodromal or mild cognitive impairment populations. Notably, we focused more intensely than other studies on determining the utility and feasibility of serum p75NTR-ECD in discriminating between AD and controls and other diseases. These findings may provide easy and more convenient access to AD diagnosis in relevant studies.

In summary, AD patients have abnormal p75NTR-ECD profiles. Knowledge of this may provide a novel perspective into AD pathogenesis. The present study indicates substantial diagnostic potential for p75NTR-ECD in AD; a longitudinal study that integrates brain imaging is warranted.

## Figures and Tables

**Table 1:** Demographic and clinical characteristics of patients in four groups (serum samples)

*Variables*	*EC,* n=*129*	*AD,* n=*156*	*PD,* n=*79*	*Stroke,* n=*83*	P-*value*
Age, years, means±s.d.	71.7±9.6	70.8±15.2	68.6±8.0	69.8±9.9	0.272
Education, years, means±s.d.	11.5±3.9	8.2±4.2	10.7±4.7	11.1±4.1	0.568
Female gender, *n* (%)	53 (41.1)	83 (53.2)	43 (54.4)	39 (47.0)	0.145
MMSE score, means±s.d.	28.0±2.0	13.6±6.7	26.0±3.8	27.0±3.8	<0.001
CDR score, means±s.d.	0.0±0.0	2.0±1.2	0.2±0.5	0.1±0.2	<0.001
ADL score, means±s.d.	20.8±2.2	48.1±19.4	23.0±6.9	32.0±8.5	<0.001
Hyperlipidaemia, *n* (%)	30 (23.2)	28 (17.9)	19 (24.1)	22 (26.5)	0.278
Hypertension, *n* (%)	55 (42.6)	54 (34.6)	23 (29.1)	32 (38.5)	0.228
Diabetes mellitus, *n* (%)	14 (10.9)	32 (20.5)	6 (8.1)	10 (12.0)	0.047
ApoEɛ4 carrier, *n* (%)	30 (23.3)	63 (40.4)	—	—	0.002
Heterozygote, *n* (%)	30 (23.3)	51 (32.7)	—	—	0.079
Homozygote, *n* (%)	0.0 (0.0)	12 (7.7)	—	—	0.001
Serum Aβ40 (pg ml^−1^), means±s.d.	60.2±34.7	86.2±55.5	—	—	<0.001
Serum Aβ42 (pg ml^−1^), means±s.d.	49.3±27.7	68.4±61.9	—	—	0.001
Serum total tau (pg ml^−1^), means±s.d.	181.0±103.2	227.1±102.2	—	—	<0.001
Serum ptau_181_ (pg ml^−1^), means±s.d.	107.1±57.8	150.8±57.70	—	—	<0.001

Abbreviations: AD, Alzheimer's disease; ADL, activities of daily living; CDR, clinical dementia rating; EC, elderly controls without neurologic disorders; MMSE, mini-mental state examination; PD, Parkinson disease.

**Table 2:** The correlation of serum p75NTR-ECD with neuropsychological evaluation score

	*Unadjusted*			*Adjusted*^a^		
	r-*value*	*95% CI*	P-*value*	r-*value*	*95% CI*	P-*value*
*MMSE*						
** **Overall	−0.472	−0.558–−0.377	<0.001	−0.482	−0.592–−0.364	<0.001
** **AD	−0.363	−0.492–−0.219	<0.001	−0.374	−0.500–−0.213	<0.001
						
*CDR*						
Overall	0.457	0.360–0.545	<0.001	0.460	0.341–−0.577	<0.001
AD	0.305	0.157–0.441	<0.001	0.304	0.142–0.453	<0.001
						
*ADL*						
Overall	0.443	0.345–0.532	<0.001	0.438	0.304–0.551	<0.001
AD	0.325	0.177–0.459	<0.001	0.320	0.160–0.453	<0.001

Abbreviations: AD, Alzheimer's disease; ADL, activities of daily living; CDR, clinical dementia rating; CI, confidence interval; MMSE, mini-mental state examination; p75NTR-ECD, ectodomain of p75 neurotrophin receptor.

a Adjusted for age, sex, education, hyperlipidaemia, hypertension and diabetes.

**Table 3:** Discriminative values of serum p75NTR-ECD using an ROC curve analysis

*ROC analysis*	*AD versus EC*	*AD versus PD*	*AD versus stroke*	*AD versus non-AD*
AUC	0.752	0.689	0.702	0.720
*P*-value	<0.001	<0.001	<0.001	<0.001
95% CI	0.695–0.808	0.623–0.754	0.633–0.771	0.666–0.774
Cutoff, pg ml^−1^	34.2	48.6	35.8	41.5
Sensitivity, %	70.5	48.7	65.4	63.4
Specificity, %	74.4	93.7	81.9	76.3
Accuracy, %	72.5	75.3	73.7	69.9

Abbreviations: AD, Alzheimer's disease; AUC, area under the ROC; 95% CI, 95% confidence interval; EC, elderly control without neurologic disorders; PD, Parkinson disease; p75NTR-ECD, ectodomain of p75 neurotrophin receptor; ROC, receiver operating characteristic.
