# Supplementary material for: Differential levels of p75NTR ectodomain in CSF and blood in patients with Alzheimer's disease: a novel diagnostic marker
Source: Transl Psychiatry. 2015 Oct 6;5(10):e650–. doi: 10.1038/tp.2015.146 (PMC4930124; doi:10.1038/tp.2015.146)
Supplement: Supplementary Information [file tp2015146x1.doc]

**Supplementary Table 1** Demographic and clinical characteristics of patients in AD and EC groups (CSF samples)

| **Variables** | **EC, n=26** | **AD, n=25** | ***p* value** |
| --- | --- | --- | --- |
| **Age, y, mean±SD** | 70.2±8.5 | 69.5±9.9 | 0.796 |
| **Education, y, mean±SD** | 11.4±3.4 | 8.52±4.6 | 0.010 |
| **Female gender, n(%)** | 10(38.5) | 13(52.0) | 0.404 |
| **MMSE score, mean±SD** | 27.7±1.8 | 12.8±6.4 | <0.001 |
| **Hyperlipidemia, n(%)** | 10(38.5) | 12(48.0) | 0.382 |
| **Hypertension, n(%)** | 11(42.3) | 7(28.0) | 0.577 |
| **Diabetes mellitus, n(%)** | 3(11.5) | 4(16.0) | 0.703 |
| **ApoEε4 carrier, n(%)** | 4(15.4) | 9(36.0) | 0.116 |
| **CSF Aβ40 (pg/ml), mean±SD** | 392.9±65.1 | 367.3±98.4 | 0.277 |
| **CSF Aβ42 (pg/ml), mean±SD** | 425.7±140.5 | 243.4±99.9 | <0.001 |
| **CSF total tau (pg/ml), mean±SD** | 325.5±104.7 | 466.8±231.2 | 0.030 |
| **CSF ptau181 (pg/ml), mean±SD** | 55.4±29.2 | 127.5±53.5 | <0.001 |

Abbreviations: AD, Alzheimer’s disease; EC, elderly controls without neurologic disorders; CSF, cerebrospinal fluid; MMSE, mini-mental state examination.

**Supplementary Table 2** The discriminatory values of serum and CSF biomarkers in AD vs EC.

| **ROC analysis** | **AUC** | ***P* value** | **95% CI** |
| --- | --- | --- | --- |
| **Serum p75NTR-ECD** | 0.752 | <0.001 | 0.695-0.808 |
| **Serum Aβ42** | 0.585 | 0.014 | 0.519-0.651 |
| **Serum Aβ40** | 0.635 | <0.001 | 0.570-0.699 |
| **Serum total Aβ** | 0.658 | <0.001 | 0.595-0.721 |
| **Serum Aβ42/40** | 0.557 | 0.097 | 0.490-0.624 |
| **Serum ptau181** | 0.702 | <0.001 | 0.642-0.763 |
| **Serum total tau** | 0.679 | <0.001 | 0.614-0.744 |
| **Serum ptau181/Aβ42** | 0.565 | 0.061 | 0.497-0.632 |
| **Serum total tau/Aβ42** | 0.515 | 0.659 | 0.447-0.584 |
| **CSF p75NTR-ECD** | 0.815 | <0.001 | 0.700-0.931 |
| **CSF Aβ42** | 0.871 | <0.001 | 0.774-0.967 |
| **CSF Aβ40** | 0.560 | 0.462 | 0.397-0.723 |
| **CSF total Aβ** | 0.803 | <0.001 | 0.684-0.922 |
| **CSF Aβ42/40** | 0.835 | <0.001 | 0.720-0.950 |
| **CSF ptau181** | 0.908 | <0.001 | 0.817-0.998 |
| **CSF total tau** | 0.678 | 0.030 | 0.525-0.830 |
| **CSF ptau181/Aβ42** | 0.974 | <0.001 | 0.940-1.000 |
| **CSF tau/Aβ42** | 0.851 | <0.001 | 0.743-0.958 |

Abbreviations: AD, Alzheimer’s disease; EC, elderly controls without neurologic disorders; AUC, the area under ROC; 95% CI, 95% confidence interval.

**Supplementary** **Table 3** The discriminatory values of serum and CSF p75NTR-ECD combining with other biomarkers in AD vs EC.

| **ROC analysis** | **AUC** | ***P* value** | **95% CI** | **Sensitivity,%** | **Specificity,%** | **Accuracy, %** |
| --- | --- | --- | --- | --- | --- | --- |
| **Serum p75NTR-ECD & serum Aβ42** | 0.772 | <0.001 | 0.718-0.826 | 62.2 | 79.8 | 71.0 |
| **Serum p75 NTR-ECD & serum Aβ40** | 0.788 | <0.001 | 0.735-0.841 | 83.3 | 57.3 | 70.3 |
| **Serum p75 NTR-ECD & serumTotal Aβ** | 0.799 | <0.001 | 0.747-0.850 | 79.5 | 69.7 | 74.6 |
| **Serum p75 NTR-ECD & serum Aβ42/Aβ40** | 0.768 | <0.001 | 0.713-0.824 | 70.5 | 76.7 | 73.6 |
| **Serum p75 NTR-ECD & serum ptau181** | 0.794 | <0.001 | 0.743-0.845 | 77.6 | 68.2 | 72.9 |
| **Serum p75 NTR-ECD & serum total tau** | 0.774 | <0.001 | 0.720-0.828 | 59.6 | 82.9 | 71.3 |
| **Serum p75 NTR-ECD & serum ptau181/Aβ42** | 0.750 | <0.001 | 0.693-0.806 | 67.3 | 74.4 | 70.9 |
| **Serum p75 NTR-ECD & serum total tau/Aβ42** | 0.754 | <0.001 | 0.698-0.810 | 60.9 | 80.6 | 70.8 |
| **CSF p75 NTR-ECD & CSF Aβ42** | 0.934 | <0.001 | 0.870-0.997 | 88.0 | 88.5 | 88.2 |
| **CSF p75 NTR-ECD & CSF Aβ40** | 0.820 | <0.001 | 0.703-0.937 | 76.0 | 84.6 | 80.3 |
| **CSF p75 NTR-ECD & CSF total Aβ** | 0.905 | <0.001 | 0.823-0.986 | 88.0 | 80.7 | 84.4 |
| **CSF p75 NTR-ECD & CSF Aβ42/Aβ40** | 0.906 | <0.001 | 0.822-0.990 | 84.0 | 92.3 | 88.2 |
| **CSF p75 NTR-ECD & CSF ptau181** | 0.942 | <0.001 | 0.875-1.000 | 100.0 | 80.8 | 90.4 |
| **CSF p75 NTR-ECD & CSF total tau** | 0.818 | <0.001 | 0.702-0.935 | 76.0 | 80.8 | 78.4 |
| **CSF p75 NTR-ECD & CSF ptau181/Aβ42** | 0.980 | <0.001 | 0.949-1.000 | 96.0 | 96.2 | 96.1 |
| **CSF p75 NTR-ECD &CSF total tau/Aβ42** | 0.888 | <0.001 | 0.793-0.983 | 80.0 | 92.3 | 86.2 |

Abbreviations: AD, Alzheimer’s disease; EC, elderly controls without neurologic disorders; AUC, the area under ROC; 95% CI, 95% confidence interval.

**Supplementary Figure 1**


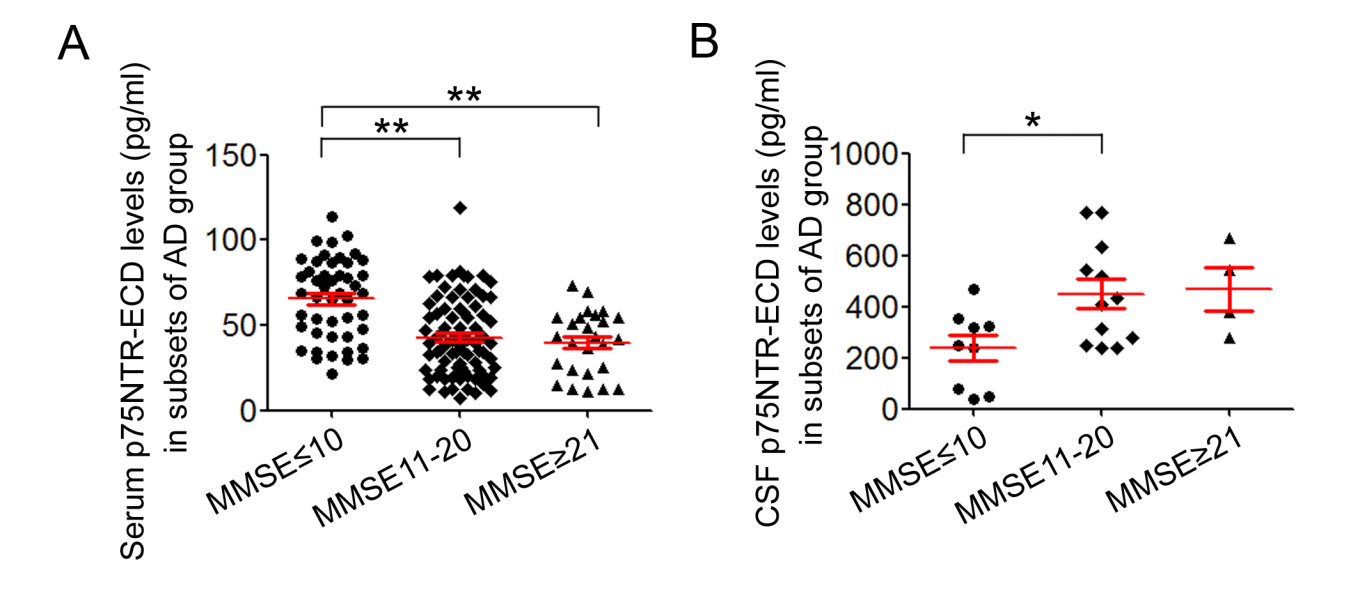


**Supplementary Figure 1** The levels of serum or CSF p75NTR-ECD in three subsets of AD patients: MMSE≤10, MMSE 11-20 and MMSE≥21. (A): Serum p75NTR-ECD concentrations in the three subsets. MMSE≤10, n=47; MMSE 11-20, n=83; MMSE≥21, n=26. (B): CSF p75NTR-ECD concentrations in the three subsets. MMSE≤10, n=9; MMSE 11-20, n=12; MMSE≥21; n=4. Mean ± S.E.M., one-way ANOVA, Tukey’s test.
